# Comparative study of polyphenol extraction using physical techniques and water as a solvent: a sustainable approach for the valorization of apple pomace

**DOI:** 10.1007/s11356-024-34637-4

**Published:** 2024-08-10

**Authors:** Silvia Fraterrigo Garofalo, Francesca Demichelis, Veronica Peletti, Lorenzo Picco, Tonia Tommasi, Debora Fino

**Affiliations:** 1 Department of Applied Science and Technology (DISAT), Politecnico Di Torino, Corso Duca Degli Abruzzi 24, 10129 Turin, TO Italy; 2 Vortex S.R.L, Via Principe Amedeo 11, 10123 Turin, TO Italy

**Keywords:** Food waste, Apple pomace, Green extraction, Ultrasound-assisted extraction, Microwave-assisted extraction, Polyphenols

## Abstract

Apples are among the most commonly cultivated fruits globally. Approximately 65% of annual apple production is transformed into apple juice concentrate generating a large amount of waste material named apple pomace, which includes seeds, skin, and other components. Disposing of apple by-products directly into the environment constitutes a source of environmental pollution due to its high-water content and easily fermentable nature. Apple pomace is rich in polyphenols that can be utilized as active components in cosmetic, nutraceutical, or pharmaceutical products. The present study aims to describe and compare different physical methods for the extraction of polyphenols from apple pomace. Water was used as the extraction solvent in thermal-stirred extraction (TSE), ultrasound-assisted extraction (UAE), and microwave-assisted extraction (MAE). The best extraction conditions were identified in terms of solid to solvent ratio, temperature, power, and time through a kinetic study. The best extraction parameters were compared environmentally on a pilot scale through a life cycle assessment (LCA). All the results demonstrated the MAE is the best technique to extract polyphenol from apple pomace in terms yield and environmental impact proving that it is possible to transform waste into a sustainable source of bioactive ingredients.

## Introduction

Apples (scientific name: *Malus Domestica*) are one of the most consumed fruits around the world, cultivated primarily in temperate regions (Shalini and Gupta 2010). In the EU, the main producers of apples are Poland, Italy, and France. The European production of apples in 2022 was estimated at approximately 12 million tonnes (European Commission). Approximately 71% of apples are consumed in their fresh state, whereas roughly 20% undergo processing to yield value-added products. Within this processed category, approximately 65% are transformed into apple juice concentrate, with the remaining portion being utilized in the production of various other items. These include packaged natural ready-to-serve apple juice, apple cider, wine, vermouth, apple purees, jams, and dried apple products (Shalini and Gupta 2010). The processing methods mentioned above result in the production of waste, specifically apple pomace, which includes seeds, skin, and other components. This waste typically accounts for 20–30% of the initial apple weight, a proportion that varies depending on the apple variety and the processing technology employed (Kumar et al. 2021a, b). Discarding these by-products directly in the environment can have several dangerous impacts as the production of greenhouse gases, and contamination of underground water table, they can represent a source of secondary pollution, such as emitting a foul smell due to microbial attack and also acting as human disease vectors (Shalini and Gupta 2010; Singh et al. 2013; Kumar et al. 2021a, b). The effective management of apple pomace requires strategic approaches to minimize the environmental impact. Thanks to its composition and ample availability, apple pomace could potentially serve as a substrate in biorefining processes. This would involve optimizing substrate conversion, minimizing energy and chemical usage, and enhancing the production of valuable added products (Kumar et al. 2021a, b; Wu et al. 2023). Apple pomace contains a large number of compounds such as polyphenols, dietary fibers, fatty acids, and minerals that can be used as active ingredients in cosmetic, nutraceutical, or pharmaceutical products (Barreira et al. 2019; Lyu et al. 2020) In particular, polyphenols are known to have the ability to counteract ultraviolet radiation-induced skin damage, photoaging, melasma, and photocarcinogenesis and have demonstrated significant antioxidant, anti-inflammatory, and immune-modulating properties. Polyphenols in apples are mainly flavonoids and phenolic acids, and research has shown that total polyphenol content (TPC) in the fresh from 5.6 to 22.1 mg GAE/g (GAE, gallic acid equivalents) depending on the cultivation species (Francini & Sebastiani 2013) and they are mainly present in the peels (Liu et al. 2021). This statement implies that apple pomace is a potential source of phenolic compounds and it can be used to develop value-added ingredients by extraction of bioactive polyphenols (Liu et al. 2021).

Liquid–liquid and solid–liquid extraction methods have historically been the most commonly employed techniques due to their user-friendliness, effectiveness, and versatile applications. These processes typically use conventional solvents such as alcohols (methanol, ethanol), acetone, diethyl ether, and ethyl acetate, often in combination with varying proportions of water. These solvents can represent potential risks to human health and can result in adverse environmental effects, as their manufacture, application, and disposal can lead to air pollution, the depletion of natural resources, and the generation of hazardous waste (Brglez Mojzer et al. 2016, Joshi and Adhikari, 2019).

Recent studies recommended the use of innovative technologies to increase the yields of bioactive compounds and to improve the efficiency of the extraction procedures while reducing the use of chemicals and energy consumption. Between the plethora of innovative technologies, ultrasound-assisted extraction (UAE) and microwave-assisted extraction (MAE), have been recently explored to extract multiple bioactive compounds from plant material (Garcia-Vaquero et al. 2020). Microwaves interact with the polar molecules of the solvent, generating a rapid and uniform heating of the matrix, while ultrasound, through the phenomenon of cavitation, enables a faster transfer of molecules from the matrix to the solvent compared to conventional methods. These two techniques enhance the extraction efficiency reducing also the extraction time (Ince et al. 2014).

In the literature, there are no studies comparing these two techniques for the extraction of polyphenols from apple pomace. The present study aims to describe and compare these two different physical methods and to identify the best conditions for the extraction of polyphenols from apple pomace. In addition, water was used as the extraction solvent. Water is low cost, it is able to solubilize several polyphenols, and it is considered the “greenest solvent” thanks to its properties such as non-toxicity, renewability, safety and ease of handling, ease of treatment, and degradation (Pingret et al. 2012; Lajoie et al. 2022). The polyphenol extraction methods were investigated to consider both the efficiency of the process, in terms of product characteristics and yield, and their effects on the environment. An environmental analysis was performed using the life cycle assessment (LCA) methodology to evaluate and compare the environmental advantages and disadvantages associated with the investigated extraction processes.

## Material and methods

### Samples

Apple pomace samples were kindly supplied by “Il Frutto permesso” agricultural cooperative in Bibiana (TO), Piedmont, Italy. The pomaces were obtained from a mixture of different regional apple “old varieties,” such as Golden Delicious, Red Delicious, Crimson, Granny Smith, and Gala varieties. The fruits were harvested during the 2022 season at different times according to the seasonality of each variety (late September for Golden, mid to late October for Crimson). Fresh apple pomace was lately obtained as by-products from on-site BIO juice production. For further operations, samples were dried by Vortex srl (Turin, Italy) using a relatively low temperature (50 °C) drying technique (under patent) to preserve the stability of bioactive compounds. Later, ground into a fine powder and stored protected from light and humidity until further analysis. The sample was then used for polyphenol extractions and did not need to be stored in the refrigerator.

### Reagents

Gallic acid and sulphanilamide used as standards were acquired from Sigma-Aldrich (St. Louis, MO, USA). Methanol, Folin-Ciocalteu reagent, sodium carbonate, sodium sulfate, and tungsten oxide were provided by Merck (Darmstadt, Germany). Water used as extraction solvent and as dilution medium during analysis was distilled water obtained from a laboratory distillation apparatus. All reagents were of analytical grade with a purity level exceeding 99%, as indicated on the labels.

### Proximate composition

The proximate composition of pomace was determined using AOAC official methods (AOAC 2019). All the analyses were performed in triplicate. Moisture was determined on sample weight loss after a night passed in the air oven at 103 °C. For ash determination, 0.5 g of dry samples were heated in a muffle furnace at 550 °C for 6 h and the residues were weighted. Lipids were determined using the AOAC Official Method 920.39, where 5 g of sample are treated with petroleum ether in a Soxhlet apparatus. Protein content was determined using the Dumas method, where a CHNS analyzer (Vario MACRO cube, Elementar Italia Srl) was used to determine the % nitrogen. This value was then multiplied by a 4.4 conversion factor to obtain the % protein (Mariotti et al. 2008). Carbohydrates were determined by difference.

### Water extraction of polyphenols

A screening design approach was adopted as an optimization strategy. For this study, a total of 27 screening experiments were conducted for each extraction method. Previous studies revealed that the extraction technique (MAE or UAE), solid-to-liquid ratio, temperature, or power were the most significant extraction parameters (Veggi et al.^2012^, Kumar et at. 2021a). Nevertheless, to gain a more comprehensive understanding of the extraction mechanisms, an additional series of experiments was carried out without the application of any electromagnetic or ultrasonic waves, i.e., subjecting samples to only stirring and heating. This set of experiments will be referred to as the “thermal-stirred extraction (TSE) set” for clarity and was performed on an electric hot plate. The duration of each test was set at a constant value of 5 min. The stirring conditions, as well as the type of solvent, were also kept constant throughout the experiments. The solid-to-liquid (S/L) ratio was varied between 1:10, 1:20, and 1:30 every 3 experiments. Each set of 3 experiments at fixed solid/liquid ratio was run at 30, 50, and 60 °C for UAE and TSE and at 200, 300, and 400 W for MAE. S/L, temperature, and power were the independent variables, the response of the experiments; in this case, the total phenolic content represents the dependent variable.

For the ultrasound-assisted extraction (UAE), water was added to appropriate volume in beakers, based on the solid-to-liquid ratio of the experiment, and a magnetic stir bar was inserted to ensure solution homogeneity during the extraction time by stirring the system. The experimental setup included an electric hot plate for temperature and stirring speed control. The diluted samples were placed inside an ultrasonic bath, positioned above the plate. The UAE equipment consisted of an ultrasonic generator (Sonics VCX 750 Ultrasonic liquid processor) connected to a probe of 13 mm which was immersed 2 cm deep into the beaker containing the test sample. The generator was configured at maximum amplitude of 40% and in a regular intervals pulse mode of 5 s ON-5 s OFF to prevent the system from overheating. The temperature inside the ultrasound bath was monitored using a temperature probe connected to the hot plate and it is set based on the screening experiment (30, 50, or 60 °C).

The microwave-assisted extraction (MAE) was carried out in a microwave digestor (Anton Paar Multiwave 5000) within a specific support instrument, Rotor 16HF100. It is a drum vessel capable of holding 16 high-pressure reaction vessels, ensuring high sample throughput and efficient handling. The reaction temperature is controlled through an IR sensor underneath the oven cavity, which measures each microwave digestion vessel through ports in the rotor base. Each dried apple pomace sample was diluted and placed in a vial body, then inserted into the vial jacket and sealed tightly with a screw cap.

At the end of each trial, the extract was filtered using folded qualitative paper filter Whatman grade 1 (particle retention 10–20 µm) (particle retention 10–20 µm) through a vacuum pump when necessary and then appropriately stored in Falcon tubes in a refrigerated environment until the determination of the total polyphenol content using the Folin-Ciocalteu method.

### Total phenolic content (TPC)

Total polyphenol content (TPC) of apple pomace samples was determined according to Folin-Ciocalteu (F–C) colorimetric method, following the standard procedure outlined in ISO 14502–1:2005. Briefly, 1 mL of diluted extract (30 mg/mL) was mixed with 5 mL of F–C reagent (10% v/v). Within 3 min to 8 min, 4 mL of sodium carbonate (7.5% w/v) was added. After incubation at ambient temperature in the dark for one hour, the absorbance at 765 nm with UV–Vis spectrophotometer DR 5000 (Hach) was measured. The results were expressed as mg gallic acid equivalent (GAE) per g of dry apple pomace (DAP). To evaluate TPC a calibration curve of gallic acid was constructed (*R*^2^ = 0.9993).

### Kinetic modeling

A set of experiments was conducted to study the extraction kinetics and analyze the time-dependent evolution of polyphenol concentration extracted from apple pomace samples under the previously identified optimal conditions. During the kinetic modeling experiments, the solvent-to-biomass ratio and temperature or power were kept constant at the optimal values obtained from the screening experiments. The extraction time was varied at specific intervals. The experimental setup was prepared for TSE, UAE, and MAE. UAE was conducted continuously for 10, 20, 30, 40, 50, 60, 120, 180, 240, 300, 600, 900, 1800, and 3600 s. The extracts were analyzed using the Folin-Ciocalteu method described in the “Total phenolic content (TPC)” section. From the obtained results, it was possible to construct the experimental kinetic curve by plotting the variation of TPC over time.

To study the kinetic of the process, Peleg’s mathematical model was used. The experimental curve showed a similar shape to the absorption curve (moisture content over time) described by the Peleg (1988). This allowed to adopt the Peleg model for describing the extraction kinetics, relating the time and the process yield in terms of TPC (Eq. 1). The kinetics of extraction was described using the following equation:

1 $$C \left(t\right)={C}_{0}+\frac{t}{{k}_{1}+{k}_{2}t}$$

where *C*(*t*) (mgGAE/gDAP) is the concentration of total polyphenols at *t* time, *K*_1_ (min gDAP/mgGAE) is Peleg’s rate constant, *K*_2_ (gDAP/mgGAE) is Peleg’s capacity constant, and *t* is the instant time at which sampling was performed and represents the extraction time, expressed in (s). *C*_0_ is the concentration of polyphenols at initial of extraction, i.e., when *t* = 0. To represent apple pomace concentration with respect to time, initial concentration was assumed equal to zero and the Peleg’s kinetic equation was modified as follows:

2 $$C \left(t\right)=\frac{t}{{k}_{1}+{k}_{2}t}$$

The Peleg’s constant rate, *K*_1_ and capacity constant, *K*_2_ are related to extraction rates at the very beginning course (*t* = 0) and equilibrium yield (*t* = 1), respectively. At the equilibrium yield, the polyphenol content is considered maximum at the condition. The Peleg’s kinetic rate constant *K*_1_ is correlated to the extraction rate (*B*_0_) at the initial time (*t* = 0) using Eq. 3:

3 $${B}_{0}=\frac{1}{{k}_{1}}$$

The Peleg’s capacity constant* K*_2_ refers to the maximum extraction yield or equilibrium concentration (*C*_*e*_) that the curve approaches as the extraction time tends to infinity. Its expression is given by Eq. 4:

4 $${\left.C\right|}_{t\to \infty }={C}_{e}=\frac{1}{{k}_{2}}$$

*K*_1_ can be determined using the plotted graph of t/C(t) vs. 1/t and *K*_2_, a capacity constant, can be determined using slope and intercept, respectively.

### Statistical analysis

All experiments were conducted in triplicates to assess the reproducibility of the values obtained at different process parameters. A one-way ANOVA test of variance was conducted to analyze the differences between means of the independent groups. Furthermore, Tukey’s test was applied for post hoc analysis to identify significant differences among multiple groups and pinpoint specific groups contributing to these differences. The confidence intervals were set to 95%, as *p* < 0.05 was considered statistically significant. The statistical analysis was conducted by the software Minitab 18.

### Life cycle assessment

After proving the technical feasibility and the optimal extraction conditions, the environmental assessment was performed with life cycle assessment (LCA). LCA was done according to ISO 14040–14044 (2006) with SimaPro 9.4 software and database Ecoinvent 3.8. The aim of the study was the evaluation and comparison of the environmental impacts associated with the three investigated types of extraction: water-based, ultrasound-assisted extraction (UAE), and microwave-assisted extraction (MAE). The scope of the study was the identification of the lowest environmentally impactful configuration to adopt at the industrial scale. Therefore, the LCA was based on technical data collected at the laboratory scale (as described in paragraph 2.4) but scaled at pilot dimension with a conversion factor in accordance with (Perry 2018). This choice was done to overcome the energy consumption limitations associated to laboratory equipment. The functional unit was 0.25 kg/d of produced polyphenol solution according to (De Luna et al. 2023), which allowed the comparison of the three extraction techniques. The study was localized in Piedmont (North-West region of Italy). The approach was from grave to gate. In accordance with De Luna et al. (2023) an attributional life cycle assessment (LCA) was adopted to determine the portion of global environmental impacts attributed to the product. The inventory data are detailed in Table 1. The polyphenol solution production system was divided into a foreground and background systems. The foreground system was directly associated with the FU and focused on the extraction process, characterized by primary data from experimental activities (described in the “Water Extraction of polyphenols” section). The background system, linked with the foreground, encompassed aspects like energy production and chemical supply (Thushari et al. 2020). For this background system, data from Ecoinvent 3.8 were adopted, including energy consumption of the equipment used in extraction experiments, based on pilot equipment specified in Table 1. The waste from the extraction process was primarily water, which was treated in a municipal wastewater treatment plant due to its non-harmful nature. The zero-burden assumption was applied to apple pomace, assuming no credits for impacts in prior lifecycle stages except for those associated with refrigerated transport to the treatment plant. Life cycle impact assessment was performed using the ReCiPe 2016 Midpoint (H) and the cumulative energy demand (CED) methods. The ReCiPe 2016 Midpoint (H) method was applied to analyze various impact categories. The focus was on the climate change impact category (kg CO_2_ eq) to understand the extraction systems’ contribution to climate change. This change is indicative of the global warming potential (GWP) resulting from greenhouse gas emissions and it is the most investigated impact category in extraction processes (Carlqvist et al. 2022). The CED accounted for non-renewable energy use throughout the lifecycle, encompassing energy consumption during the transportation of apple pomace and the polyphenol extraction process. A sensitivity analysis was carried out by varying polyphenol yield production by ± 5% w/w, to prove the potential variations in the environmental impact response of the investigated extraction systems.

**Table 1:** The inventory data for the three extraction techniques, providing specific information about each step of the process, including the flow and the source of the data

	Water-based extraction				
**Step 1: collection and transport**	**Input**	Amount	unit	Ecoinvent	Reference
	Apple pomace	30.86	kg/d	/	Primary data
	Refrigerated transport	10	km	Transport, freight, lorry with refrigeration machine, 3.5–7.5 ton, EURO4, R134a refrigerant, freezing {GLO}| transport, freight, lorry with refrigeration machine, 3.5–7.5 ton, EURO4, R134a refrigerant, freezing | Cut-off, S	Primary data
	**Output**				
	Transported apple pomace	30.86	kg/d		
**Step 2: water extraction**	**Input**				
	Transported apple pomace	30.86	kg/d		
	Water	308.64	kg/d	Water, cooling, drinking,	Primary data
	Energy for the reactor Glass and Stainless Lab Scientific	1.05	kWh/d	Electricity, high voltage {IT}| market for | Cut-off, S	Glass & Stainless Steel Reactors | Labfirst Scientific (lab1st.com)
	**Output**				
	Polyphenol solution	0.25	kg/d		Primary data
	Wastewater	339.25	kg/d	Waste water, water disposal, wastewater, average Europe without Switzerland	Secondary data
	**Ultrasound-assisted extraction**				
**Step 1: collection and transport**	**Input**	Amount	unit	Ecoinvent	Reference
	Apple pomace	24.53	kg/d	/	Primary data
	Refrigerated transport	10	km	Transport, freight, lorry with refrigeration machine, 3.5–7.5 ton, EURO4, R134a refrigerant, freezing {GLO}| transport, freight, lorry with refrigeration machine, 3.5–7.5 ton, EURO4, R134a refrigerant, freezing | Cut-off, S	Primary data
	**Output**				
	Transported apple pomace	24.53	kg/d		
**Step 2: ultrasound-Assisted Extraction**	**Input**				
	Transported apple pomace	24.53	kg/d		
	Water	736.01	kg/d	Water, cooling, drinking	Primary data
	Energy for the Ultrasonic-homogenizers Hielscher	2.77	kWh	Electricity, high voltage {IT}| market for | Cut-off, S	https://www.hielscher.com/ultrasonic-homogenizers-for-liquid-processing-3.htm?gclid=CjwKCAjwnOipBhBQEiwACyGLulAXmgKWztFDoY4Bv8HGqyKKb3h7CwP5P4zkpVqhK7JKyP2T--fHyxoChlkQAvD_BwE
	**Output**				
	Polyphenol solution	0.25	kg/d		Primary data
	Wastewater	760.29	kg/d	Waste water, water disposal, wastewater, average Europe without Switzerland	Secondary
**Microwave-assisted extraction**					
**Step 1: collection and transport**	**Input**	Amount	Unit	Ecoinvent	Reference
	Apple pomace	18.12	kg/d	/	Primary data
	Refrigerated transport	10	km	Transport, freight, lorry with refrigeration machine, 3.5–7.5 ton, EURO4, R134a refrigerant, freezing {GLO}| transport, freight, lorry with refrigeration machine, 3.5–7.5 ton, EURO4, R134a refrigerant, freezing | Cut-off, S	Primary data
	**Output**				
	Transported apple pomace	18.12	kg/d	/	/
**Step 2: microwave-Assisted Extraction**	**Input**				
	Transported apple pomace	18.12	kg/d	/	/
	Water	362.32	kg/d	Water, cooling, drinking	Primary data
	Energy for the reactor schaeferScientific	1	kWh/d	Electricity, high voltage {IT}| market for | Cut-off, S	LABOTRON HTE—Forno a microonde per applicazioni di riscaldamento R&D fino a 1600 °C—Da 3 e 6 Kw—Schaefer Italy (schaefer-tec.it)
	**Output**				
	Polyphenol solution	0.25	kg/d	/	Primary data
	Wastewater	380.18	kg/d	Waste water, water disposal, wastewater, average Europe without Switzerland	Secondary data

## Results

### Proximate composition

The proximate composition of apple pomace was determined as described in the “Proximate composition” section. Table 2 presents the results regarding the moisture, protein, carbohydrate, lipid, and ash content. All values are expressed as percentages. Since the analyses were conducted in triplicate, the values refer to the mean over the three samples, and the standard deviation is reported.

**Table 2:** Proximate composition of Apple Pomace: moisture, protein, carbohydrate, lipid, and ash content

Proximate composition of apple pomace	Mean value (%)	±
**Moisture content** (% w/w, wet AP basis)	50,45	0,21
**Proteins** (% w/w, dry AP basis)	3,14	0,20
**Carbohydrates** (% w/w, dry AP basis)	42,63	0,311
**Lipids (**lipid content (% w/w, dry AP basis))	1,75	0,05
**Ashes** (% w/w, dry AP basis)	2,09	0,05

The obtained values align closely with literature findings regarding the percentage composition, as evidenced by the data presented in Table 3.

**Table 3:** Proximate composition of Apple Pomace data from the literature (Hobbi et al. 2021) (Nayak et al. 2020) (Iqbal et al. 2021)

Proximate composition	%
Moisture	49–85
Proteins	2.31–6.98
Carbohydrates	51.1–84.7
Lipids	1.29–8.18
Ashes	0.56–4.29

### Screening experiments

**Fig. 1:**
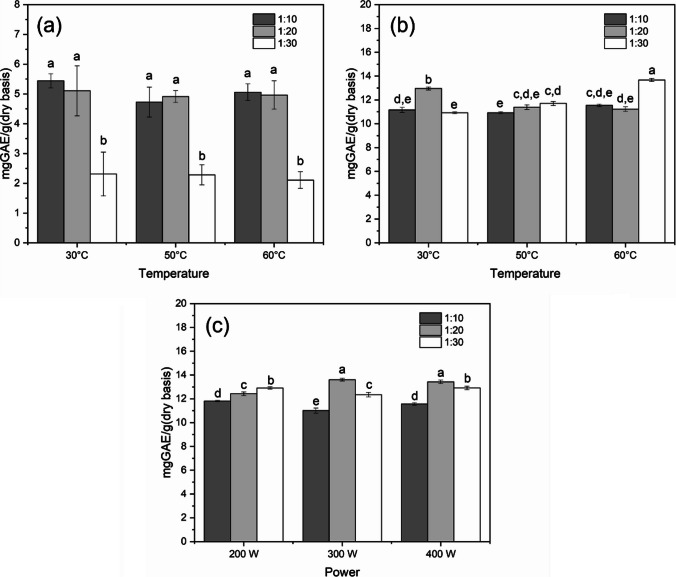
Screening experiments at different solid to solvent ratio and temperatures for TSE (**a**), UAE (**b**), and solid to solvent ratio and power for MAE (**c**). Different letters indicate differences between TFC levels significant at *p* < 0.05

Screening experiments showed that the tested extraction factors (i.e., extraction technique, temperature or power, and solvent-to-solid ratio) significantly affected the TPC in the extracts. Each set of three experiments was considered a single experimental group and classified with letters from A to I. Mean values of Total Phenolic Content (mgGAE/gDAP) and standard deviations were recorded for each group and the respective experimental conditions, including the solid-to-liquid ratio and temperature for UAE and TSE, and solid-to-liquid ratio and power for MAE are reported in Fig. 1.

Figure 1a shows that heating by thermal convection using a simple an electric hot plate generates yield lower than sample treated with US or MW at every temperature or biomass to solvent ratio. It is possible to note that yield remains constant at each temperature, but it decreases with the increase of solvent ratio Probably because in such a short time (5 min), it is difficult to uniformly heat the entire solution. The higher yield obtained by UAE compared to TSE is due to the ability of US to disrupt plant cell walls by using the cavitation phenomenon. At the same time, the cavitation effect can also increase diffusion and solubility of polyphenol (Qin et al. 2023). Throughout the compression cycle, the bubbles undergo compression, leading to an elevation in both temperature and pressure. Consequently, the bubble collapses, generating a “shock wave” that propagates through the solvent, thereby promoting enhanced mixing. Ultrasound additionally exerts a mechanical influence, as the implosion of cavitation bubbles in proximity to cell walls causes substantial cell disruption and alters the structure of plant tissue. This process facilitates the diffusion of inner cell contents into the surrounding medium (Albu et al., 2004). Figure 1b shows that for UAE the best extraction conditions are solid-to-liquid ratio of 1:30 and temperature of 60 °C. The temperature seems to have a positive effect on the yield of polyphenols. High temperature increases the disruption of the cellular structure of the matrix, increasing the permeability of the cell membrane Allowing the polyphenols to interact more effectively with the solvent by accelerating mass transfer (Mircea et al. 2020). At the same time surface tension and the viscosity decreases a enhancing wetting of the plant material resulting in a more efficient extraction. These statements are confirmed by Mircea et al. (2020) who extracted polyphenols from propolis using UAE. Also, Abi-Khattar et al. (2022) observed experimentally that an increase of 10 °C promoted the interaction between the solvent and the matrix Highlighting the significance of both mechanical damage and heating, which can lead to softening of the plant matrix, thereby enhancing the impact of cavitation. Also, Machado et al. (2019), who extracted phenolic compounds from pomegranate peel, obtained the highest antioxidant capacity at temperatures around 60 °C stating that, high temperatures can improve the breakdown of the interactions between the solute and the plant. It is important to know that temperature can be increased up to a maximum beyond which compounds can be degrade. Machado et al. (2019), suggest to not perform extraction above 60 °C. The higher yield obtained at solid-to-liquid ratio of 1:30, suggest that a larger volume of solvent helps to accelerate the diffusion process (Medina-Torres et al. 2017). These results were confirmed by Ez Zoubi et al. (2021) which extracted phenolic compounds from *Moroccan Lavandula stoechas* by UAE obtaining the best results at solid-to-liquid ratio of 1:30. Good results in yield was showed also at 30 °C and solid-to-liquid ratio of 1:20 suggesting an interaction between temperature and solid-to-liquid ratio at low temperature. This behavior was not descripted in any other works in literature and further investigation are necessary.

MWE (Fig. 1c) shows the best results in terms of yield. MW generate molecular dipole rotation, causing a swift dielectric heating of the polar components within the plant matrix and the solvent, consequently enhancing extraction kinetics and reducing extraction time (Bouchez et al. 2023a, b). The best conditions were solid-to-liquid ratio of 1:20 and power of 300 and 400 W for MAE. It was reported that the increase in irradiation power affect the extraction solvent changing its polarity, viscosity, and surface tension, leading to an increased release of solutes from plant cells (Pavlić et al. 2023). Belwal et al. (2020) confirmed these results. They extracted polyphenols from Berberis roots using MW and they noted that the TPC increased with increasing microwave power obtaining a maximum at 300 W. Pavlić et al. (2023) affirming that higher irradiation power can accelerate molecular movement and internal diffusion, resulting in the degradation of the plant material increasing the penetration of the extraction solvent increasing the mass transfer for internal diffusion from the solid to the liquid phase. Regarding the solid-to-liquid ratio from Fig. 1 it is possible to note that there is an increase in TPC from solid-to-liquid ratio of 1:10 to 1:20 and then the yield decrease again at 1:30. This decrease in polyphenol yields was explained by Vidal et al. (2015), briefly water used for extraction efficiently absorbs microwave energy, promoting increased food material swelling, thereby favorably enhancing the contact surface area between phases. However, an excessive solvent volume may diminish material microwave absorption, as it absorbs more energy. In such circumstances, the disruption of cell wall material and mass transfer could have an adverse impact on both phenolics extraction and antioxidant capacity, leading to a decrease (Vidal et al. 2015).

### Extraction kinetic

The optimal extraction time was evaluated for TSE, UAE, and MAE according to the results obtained from screening experiments. The experimental kinetic curves were obtained for each extraction method by plotting the average TPC value among the three examined samples against time. The obtained constants of Peleg’s model (rate constant *K*_1_, capacity constant *K*_2_) and calculated parameters, i.e., regression coefficient (*R*^2^), initial extraction rate (*B*_0_), and extraction extent (*C*_e_), are shown in Table 4.

**Table 4:** Peleg’s parameters for TSE, UAE, and MAE. Rate constant *K*_1_, constant capacity *K*_2_, initial extraction rate (*B*_0_), maximum yield extraction (*C*_*e*_), and regression coefficient (*R*^2^)

	***K***_**1**_** [s ∙ g DAP/mgGAE]**	***K***_**2**_** [gDAP/mgGAE]**	***B***_**0**_** [mgGAE /gDAP ∙ s]**	***C***_**e**_** [mgGAE /gDAP]**	***R***^**2**^
TSE	10.037	0.093	0.099	10.718	0.957
UAE	12.286	0.078	0.081	12.870	0.934
MAE	2.017	0.075	0.496	13.351	0.934

**Fig. 2:**
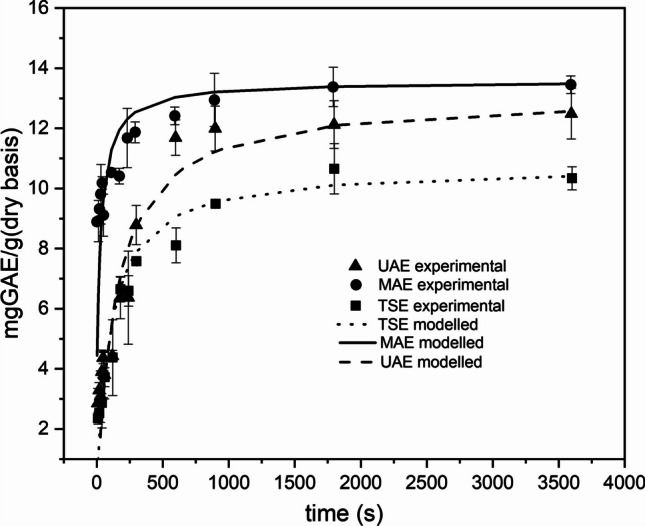
TSE, UAE, and MAE kinetic curve. Black squares, circles, and triangles represent the experimental values. Each experiment was conducted in triplicate, and the error bars correspond to standard error. Lines represent the modeled values by Peleg’s equation

For TSE, since no difference was observed between the temperatures and the 1:10 and 1:20 ratios, it was chosen to work under the most energy-efficient conditions. Consequently, the kinetics were conducted at 30°C with a 1:20 ratio in order to save water as well. As can be seen from Fig. 2 and Table 4, the maximum yield reaches approximately 10,7 mgGAE /gDAP, reaching a plateau at around 10 min.

The screening results for UAE led to the optimal conditions of a solid-to-liquid ratio of 1:30 and a temperature of 60 °C. A rapid increase in extracted TPC was observed at the beginning of the process. The curve exhibits a steeper slope during the initial 4 min of extraction, followed by a slight decline at *t* = 300 s. Within the first 10 min (600 s), more than 90% of the maximum TPC was already extracted. The maximum yield reaches approximately 12.9 mgGAE /gDAP. These results are confirmed by Vidal et al. (2015) and Galgano et al. (2021) which extracted polyphenols from almond skin by-products and lentil seed coat respectively using UAE.

The screening results for the MAE led to the optimal conditions of a solid-to-liquid ratio of 1:20 and a power of 300 W. Total extracted polyphenol content significantly increases in the initial stages and reaches a plateau after the first 240 s. The maximum TPC yield is around 13.3 mgGAE/gDAP. These results are in line with those of Pavlić et al. (2023). They applied MWE for the recovery of peppermint polyphenols and reported that a rapid extraction (up to 5 min) was sufficient for the elution of the majority of all compounds from the plant material.

It is important to mention that subjecting the sample to extended microwave exposure may result in the possible degradation of certain polyphenolic compounds (Pavlić et al. 2023).

The temporal evolution of the three extraction exhibits a hyperbolic trend, revealing that the total extracted polyphenol content significantly increases in the initial stages and reaches a plateau after a certain time. A rapid increase in extracted TPC was observed at the beginning of the process.

This behavior can be explained based on Fick’s law (Khandare et al. 2021). According to Fick’s law, at the beginning of an extraction process, high concentration gradient between the solid phase (AP) and liquid phase (water) results in high diffusion of polyphenolic compounds into the solvent. As the extraction continues, the concentration gradient gets smaller; thereby increasing the extraction yield until a peak point is attained. Khandare et al. (2021) also demonstrated that the extracted TPC was reduced beyond the peak point in all conditions. This observation may be attributed to the oxidation and decomposition of TPC leading to thermal degradation of polyphenolic compounds with sustained heating and long extraction time. This statement is consistent with literature since polyphenol stability, is recognized as a challenge for the food industry and is sensitive to temperature.

The high regression coefficients (*R*^2^ > 0.93) in all the studied conditions and corresponding graphs indicate good agreement between experimental values and predicted values calculated using Peleg’s equation proving well fit of this model. The Peleg model has been used previously to extract polyphenols from Soya waste, potato peels, and mustard seed (Khandare et al. 2021) (Kumari et al. 2017) obtaining a good fit (*R*^2^ close to 1).

Peleg’s equation frequently serves as a robust kinetic model for depicting the extraction of polyphenols from natural sources. This is due to the intricate nature of these natural matrices and the diverse array of polyphenols they contain, making it challenging to devise practical theoretical models. Furthermore, the mass transfer from the solid to the liquid phase generally follows kinetics that are slower than a first-order system (Pettinato et al. 2019).

### Life cycle assessment

A life cycle assessment (LCA) was performed to assess the environmental impact of extracting 0.25 kg/d of polyphenol solution. The environmental impacts were assessed using the ReCiPe 2016 MidPoint (H) and cumulative energy demand (CED) methods. The results for the total impact categories for the three extraction techniques, calculated using ReCiPe 2016 MidPoint (H), are presented in Table 5.

**Table 5:** The environmental impacts of all the impact categories calculated with ReCiPe MidPoint (H) for all three extraction techniques

**Impact category**	**Unit**	**Water**	UAE	MAE
Global warming	kg CO_2_ eq	6.93E-01	1.28E + 00	5.26E-01
Stratospheric ozone depletion	kg CFC11 eq	4.08E-07	8.06E-07	3.18E-07
Ionizing radiation	kBq Co-60 eq	6.55E-02	1.46E-01	5.37E-02
Ozone formation, human health	kg NOx eq	1.58E-03	2.49E-03	1.13E-03
Fine particulate matter formation	kg PM2.5 eq	7.35E-04	1.35E-03	5.56E-04
Ozone formation, terrestrial ecosystems	kg NOx eq	1.61E-03	2.54E-03	1.15E-03
Terrestrial acidification	kg SO_2_ eq	2.07E-03	3.93E-03	1.59E-03
Freshwater eutrophication	kg P eq	1.64E-04	3.09E-04	1.25E-04
Marine eutrophication	kg N eq	1.19E-05	2.21E-05	9.07E-06
Terrestrial ecotoxicity	kg 1.4-DCB	2.31E + 00	2.40E + 00	1.45E + 00
Freshwater ecotoxicity	kg 1.4-DCB	1.45E-02	1.99E-02	9.90E-03
Marine ecotoxicity	kg 1.4-DCB	1.99E-02	2.74E-02	1.36E-02
Human carcinogenic toxicity	kg 1.4-DCB	2.34E-02	3.71E-02	1.68E-02
Human non-carcinogenic toxicity	kg 1.4-DCB	3.09E-01	5.39E-01	2.29E-01
Land use	m^2^a crop eq	1.78E-02	3.09E-02	1.32E-02
Mineral resource scarcity	kg Cu eq	9.55E-04	1.32E-03	6.52E-04
Fossil resource scarcity	kg oil eq	2.13E-01	3.95E-01	1.62E-01
Water consumption	m^3^	3.18E-01	7.56E-01	3.70E-01

In this environmental evaluation, the focus was on climate change (measured in kg CO_2_ eq./FU) and energy demand (measured in MJ eq./FU). This emphasis is in line with the new target of achieving net-zero carbon emissions by 2050, as outlined in the Green Deal Europe.

**Fig. 3:**
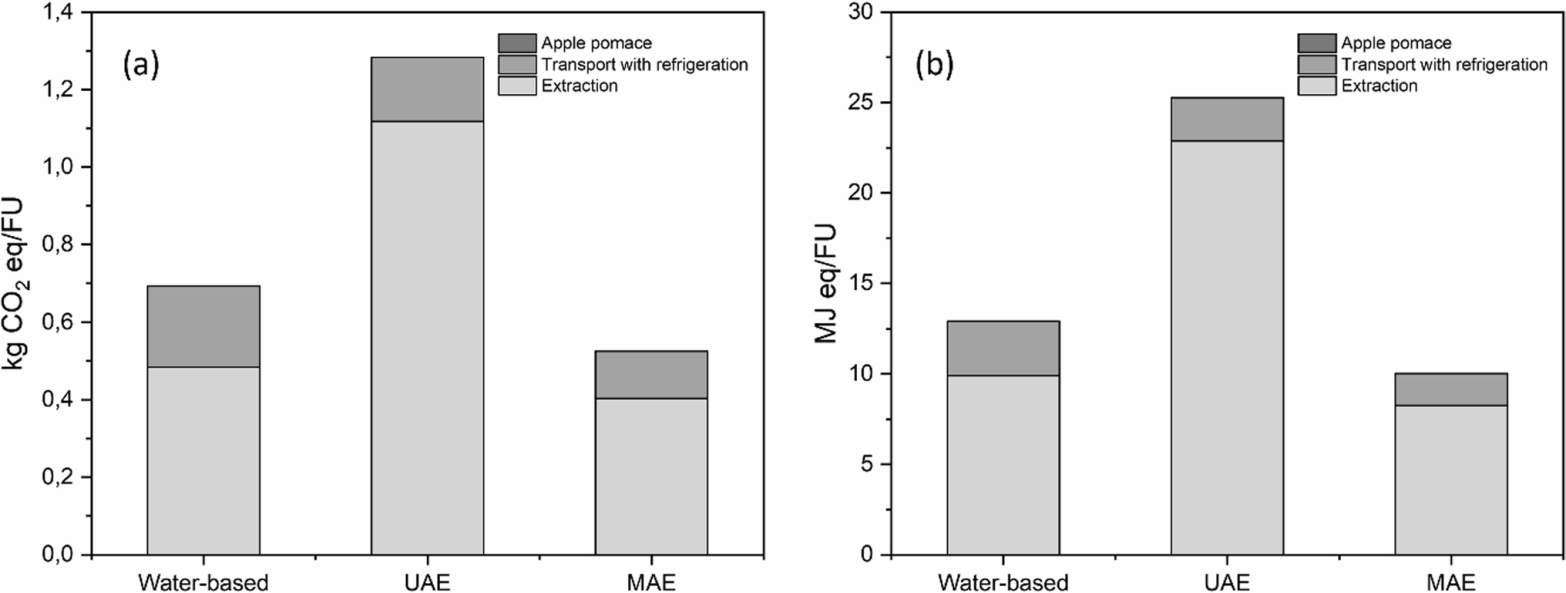
The climate change and energy demand of the three extraction techniques with the detail of the impact of the process item

Figure 3 depicts that all three extraction techniques exhibit similar trends in both the climate change category and energy demand.

Regarding the climate change impact category, the lowest environmental impact was observed with MAE, measuring 0.56 ± 0.01 kgCO_2_ eq./FU, followed closely by water-based extraction at 0.69 ± 0.03 kgCO_2_ eq./FU, and with UAE registering the highest impact at 1.28 ± 0.05 kgCO_2_ eq./FU.

In the energy demand category, there was a more evident differentiation between MAE and water-based extraction. Here, MAE demonstrated the lowest impact at 10.03 ± 0.08 MJ eq./FU, followed by water-based extraction at 12.93 ± 0.1 MJ eq./FU, with UAE having the highest impact at 25.27 ± 0.12 MJ eq./FU.

UAE extraction exhibited the highest environmental impact for two main reasons. The first is that the process involved a higher temperature (60 °C), which exceeded the temperatures used in water-based extraction and MAE. The second is that the UAE method employed a higher apple pomace to water ratio (1:30) compared to water-based and MAE extraction. The increased apple pomace to water ratio resulted in a bigger reactor working volume and more wastewater production when comparing the same functional unit. Although UAE achieved higher polyphenol yields compared to water-based extraction, the elevated temperature and the increased water consumption resulted in higher environmental impacts.

In this study, the environmental impacts were lower compared to some studies previously conducted in the literature, which examined the environmental evaluation of polyphenol extraction using UAE and MAE techniques from various sources such as pine needles (De Luna et al. 2023), rice bran (Fraterrigo Garofalo et al. 2023), sugar beet seed (Bouchez et al. 2023a, b), and spruce bark (Carlqvist et al. 2022).

In these studies, the common approach was the combination of physical treatments like UAE and MAE with solvents such as acetone (De Luna et al. 2023), isopropanol (Fraterrigo Garofalo et al. 2023), and ethanol (Bouchez et al. 2023a, b and Carlqvist et al. 2022).

As demonstrated by Carlqvist et al. (2022), the primary contributor to the overall environmental impact in the extraction process is the choice of solvent, which typically accounts for approximately 70% of the total impact when employed. The great advantage of the present study is the replacement of solvent with water, which could be further recycled to reduce the environmental impact according to De Luna et al. (2023).

Additionally, the selection of the electricity production system plays a significant role in the analysis, serving as the second most influential factor.

The results confirm that energy consumption plays a central role in the environmental profile of the three extraction techniques. Therefore, opting for an energy source with a lower proportion of energy derived from fossil fuels can reduce the environmental impacts of the extraction products. In the present study, the chosen energy was the Italian energy grid mix, and the detail of the energy source is reported in Table 6. To reduce energy consumption, a recovery energy system could be further considered according to Barjoveanu et al. (2020).

**Table 6:** The energy source employed for the three extraction techniques

Impact category	Unit	Water-based	UAE	MAE
Non-renewable, fossil	MJ	9.74	18.06	7.40
Non-renewable, nuclear	MJ	1.21	2.73	1.00
Non-renewable, biomass	MJ	0.00	0.00	0.00
Renewable, biomass	MJ	0.22	0.49	0.18
Renewable, wind, solar, geothe	MJ	0.52	1.19	0.43
Renewable, water	MJ	1.24	2.79	1.01
Total	MJ	12.93	25.27	10.03

These findings highlight the potential for more sustainable and environmentally friendly practices in the extraction of phenolic solutions from apple pomace, with MAE emerging as the most environmentally friendly option among the three technologies considered.

## Conclusions

In this study solvent extraction, ultrasound-assisted extraction and microwave-assisted extraction using water as the extraction solvent were screened to identify the best condition in terms of solid to solvent ratio, temperature, and power for the total polyphenol extraction. Once the optimal operating conditions were identified, a kinetic study was conducted to determine the extraction time for each extraction technique. For the TSE the best extraction yield is achieved at 30 °C, a solid-to-solvent ratio of 1:10, and after 10 min. For the UAE, the optimal extraction yield is attained at 60 °C, a solid-to-solvent ratio of 1:30, and after 10 min, while for the MAE, the best conditions were 300 W, 1:20, and 5 min. Then, these three technologies were compared from and environmental point of view at pilot-scale production context. The study highlighted three noteworthy findings. The first is that MAE exhibited the lowest environmental impact among all the investigated impact. The second one is that the replacement of solvent with water reduce the impacts of all the investigated techniques. The last one is the necessity to recover the energy consumed.

## Data Availability

The data can be available on request.
